# Electrogastrogram-Derived Features for Automated Sickness Detection in Driving Simulator

**DOI:** 10.3390/s22228616

**Published:** 2022-11-08

**Authors:** Grega Jakus, Jaka Sodnik, Nadica Miljković

**Affiliations:** 1 Faculty of Electrical Engineering, University of Ljubljana, 1000 Ljubljana, Slovenia; 2 School of Electrical Engineering, University of Belgrade, 11000 Belgrade, Serbia

**Keywords:** automated vehicle, electrogastrography, entropy, driving simulator, machine learning, motion sickness, nausea, noise reduction, random forest

## Abstract

The rapid development of driving simulators for the evaluation of automated driving experience is constrained by the simulator sickness-related nausea. The electrogastrogram (EGG)-based approach may be promising for immediate, objective, and quantitative nausea assessment. Given the relatively high EGG sensitivity to noises associated with the relatively low amplitude and frequency spans, we introduce an automated procedure comprising statistical analysis and machine learning techniques for EGG-based nausea detection in relation to the noise contamination during automated driving simulation. We calculate the root mean square of EGG amplitude, median and dominant frequencies, magnitude of Power Spectral Density (PSD) at dominant frequency, crest factor of PSD, and spectral variation distribution along with newly introduced parameters: sample and spectral entropy, autocorrelation zero-crossing, and parameters derived from the Poincaré diagram of consecutive EGG samples. Results showed outstanding robustness of sample entropy with moderate robustness of autocorrelation zero-crossing, dominant frequency, and its median. Machine learning reached an accuracy of 88.2% and revealed sample entropy as one of the most relevant and robust parameters, while linear analysis highlighted spectral entropy, spectral variation distribution, and crest factor of PSD. This study clearly indicates the need for customized feature selection in noisy environments, as well as a complementary approach comprising machine learning and statistical analysis for efficient nausea detection.

## 1. Introduction

Driving simulators are used in various industries to study human behavior, observe their performance and driving skills, and validate new human–machine interfaces in vehicles. They provide a robust, safe, and controllable testing environment, but sometimes cause simulation sickness and other unpleasant sensations [1,2]. The general approach to assessing sickness incorporates both subjective techniques mainly based on questionnaires (such as Motion Sickness Questionnaire or Simulator Sickness Questionnaire) and objective measures commonly comprising physiological recordings (heart rate, body temperature, electrodermal activity, electrogastrogram (EGG), etc.) [3,4,5,6]. The main difference between those two approaches is that subjective measures (driver’s perception) are administered commonly after the recording session, while physiological measurements can be used for continuous assessment. Although different in nature, for complete practical assessment, a holistic approach comprising both objective and subjective measures is advised [7,8,9].

Real time or quasi real time continuous assessment with physiological measures is an attractive approach for the evaluation of nausea and simulator sickness phenomenon. However, these measures proved to be prone to a variety of factors either due to the environmental or noise contamination as it has been shown previously for skin temperature, EDA, and EGG [9,10]. Although the measurement of pupil diameter and pupillary rhythm present an alluring non-contact method and promising approach to study not just sickness, but also emotional responses and cognitive load [11,12], these measures are not directly related to the stomach activity. Moreover, pupil diameter either measured by the eye tracker or infrared camera may have limited measurement agreements [13].

The symptoms of motion sickness include nausea, vomiting, sweating, eyestrain, difficulty focusing, headaches, oculomotor disturbances, disorientation, dizziness, vertigo, and others [14,15,16,17,18]. The most widely reported symptom is nausea [19,20]. Electrogastrogram is used for measuring gastric myoelectrical activity, and as such, it can be used to indirectly assess motion sickness, while it assesses nausea in a more direct manner as suggested by previous extensive research [14,15,16,17,18]. In this paper, we focused mainly on nausea occurrence as a result of simulator sickness being associated with gastric dysrhythmias that can be recorded via electrogastrogram [21]. An EGG-based approach for evaluating nausea occurrence presents a simple, efficient, continuous, and quantitative approach. Parameters derived from the EGG (e.g., mean amplitude, dominant frequency) and their effectiveness in nausea assessment can be observed, among others, in automated driving simulators. EGG amplitude and frequency content are altered as a consequence of nausea.

The most common changes in EGG signal caused by nausea occurrence are the frequency shift towards higher frequency ranges and amplitude increase. Specifically, the EGG signal loses its regularity (rhythm) and becomes more random in the course of nausea manifestation. EGG-derived features such as change of spectral power percentage in frequency ranges, dominant frequency, power of dominant frequency, and averaged amplitude with several less typical features such as crest factor of power spectrum and median frequency are already proposed to assess nausea-related EGG waveform alterations [4,10]. Typically, a combination of these features is used to study EGG changes caused by nausea elicitation. We calculate these customary parameters and at the same time propose novel features to reflect on overall nausea-related changes in EGG. Namely, we propose the adoption of the level of randomness (entropy), autocorrelation zero-crossing, and features derived from the Poincaré plot of consecutive EGG samples in time domain to quantify the self-similarity of the EGG time series and to detect nausea in noisy environments.

Our main goal is to examine in detail methodological aspects of EGG-based parameter usability. This comes with the unique terms of inspecting parameters’ robustness to different noise levels as well as with the combined approach comprising both classical statistical analysis and the machine learning technique as proposed earlier for studying nausea with infra-red cameras [12]. Moreover, we introduce completely novel features for studying nausea occurrence (Poincaré plots and entropy). This research could possibly complement current efforts to standardize EGG technique and to make EGG-based evaluation widely adopted for nausea assessment, as well as for studying complex brain–gut interactions [22].

### 1.1. Rationale for Introduction of New EGG-Based Parameters

The level of randomness is a particularly useful measure in many areas of data analysis. For example, in stock market analysis, approximate entropy as a measure of randomness suggests more data predictability in times of crisis indicating more pronounced repeated patterns [23]. Sample entropy is also a promising method for determining the regularity of signals based on the existence of patterns. The method is similar to approximate entropy, but it is independent of the signal length and has better relative consistency [24]. It was successfully used, for example, to separate uterine electromyogram (EMG) records of term and pre-term delivery groups [25]. On the other side, spectral entropy or the entropy of a signal normalized power distribution can be used to estimate the uniformity of signal power distribution and, as a result, discriminate between narrowband and wideband signals. The method was, for example, used to determine the depth of anesthesia from an electroencephalogram (EEG) [26,27]. For the EGG-based detection of nausea, we used both sample and spectral entropy as we hypothesize that these features would discriminate between less and more random changes in EGG signals corresponding to the baseline recording and nausea occurrence, respectively. In fact, this could fit perfectly into EGG-based nausea assessment as repeated patterns are more pronounced in the baseline EGG waveform shape consisting mainly of normal gastric rhythm and revealing narrowband nature in comparison to the EGG signal during nausea occurrence.

The autocorrelation zero-crossing is another method for determining the randomness of signals. The method is based on calculating the first zero-crossing of the autocorrelation function: the closer it is to the maximum of the autocorrelation function, the more random the signal. This method has been used successfully in the analysis of the EMG and electrocardiogram (ECG) where it showed less proneness to the signal interferences [25,28]. For that reason, we hypothesize that it could be simply adopted for EGG-based analysis.

Previously, EGG frequency dynamics were expressed by utilizing the number of turning points as a test of randomness to study gastric coupling in animals [29,30]. Here, we adopt a common approach to examine heart rate variability based on standard deviations of a Poincaré plot to study EGG-related nausea alterations. A Poincaré plot allows for the evaluation of non-linear aspects of signal sequences, and it is commonly used in biomedical engineering to characterize heart rate variability [31]. Our hypothesis for the adoption of a Poincaré plot and its adaptation in EGG analysis is based on the premise that changes in EGG signal as a result of nausea occurrence would express more non-linear properties in comparison to the baseline EGG recordings.

### 1.2. Noise Effect on EGG-Based Parameters

Although promising, EGG-based assessment of nausea comes with a major drawback—exaggerated noise. Due to the relatively low amplitude and low frequency content, the EGG signal is easily affected by noises and artifacts, especially by the movement artifacts. This is clearly pronounced in motion-based driving simulators as they come with an interactive and dynamic environment causing EGG data quality to decrease due to the excessive noise [10].

To test the applicability of previously used and newly introduced EGG-based parameters and their robustness to noise levels, we added synthetic colored noise on datasets comprising EGG signals recorded in 20 healthy participants during driving simulation to create semi-synthetic datasets. To the best of our knowledge, this is the first study reporting effect of noise on EGG features for simulator sickness-related nausea assessment.

Moreover, we introduce and demonstrate a new machine learning approach to automatically evaluate nausea occurrence by EGG-based parameters in relation to noise. We report results of both statistical analysis and machine learning by reasoning for a middle ground approach between “data modeling” and “algorithmic modeling” cultures to yield a strong empirical foundation as recommended in [32].

### 1.3. Aims of the Study

Our objective is twofold. We firstly aim at the extensive exploration of known and novel EGG-based parameters for nausea assessment. Secondly, we seek features with proven higher levels of robustness to noises and artifacts that would be more appropriate for nausea detection in dynamic driving simulator environments. To achieve our aims, we employ automated techniques for the extraction of proposed EGG-based features with various Signal-to-Noise Ratios (SNRs) with added synthetic noise and contribute to the existing body of knowledge in the following ways:

1. We present an extended list of EGG-based features for nausea assessment following pertinent reasoning for their calculation.
2. We report on the sensitivity/robustness of the proposed EGG-based parameters to different levels of SNRs and the noise effect on nausea detection.

## 2. Materials and Methods

We introduce an extensive list of EGG-based parameters for nausea detection and for the evaluation of parameter robustness to different levels of SNRs. We use both traditional statistical linear analysis and non-linear machine learning to test the usability of the selected EGG features for the detection of nausea incidence.

### 2.1. Available EGG Data and Recording Procedure

The data for the analysis are obtained from the study by Gruden et al. [4]. In the continuation, we summarize the most important information on the data collection required to explain the main goals of the present research.

The study was conducted at the Faculty of Electrical Engineering, University of Ljubljana, Slovenia. Twenty individuals (two females), mostly students or staff from the Faculty of Electrical Engineering in Ljubljana participated in the study. The participants were between 19 and 40 years old, had a valid driving license, and had more than one year of driving experience. They were instructed to fast for at least 6 h and not to drink for at least 2 h before the study [33].

**Figure 1:**
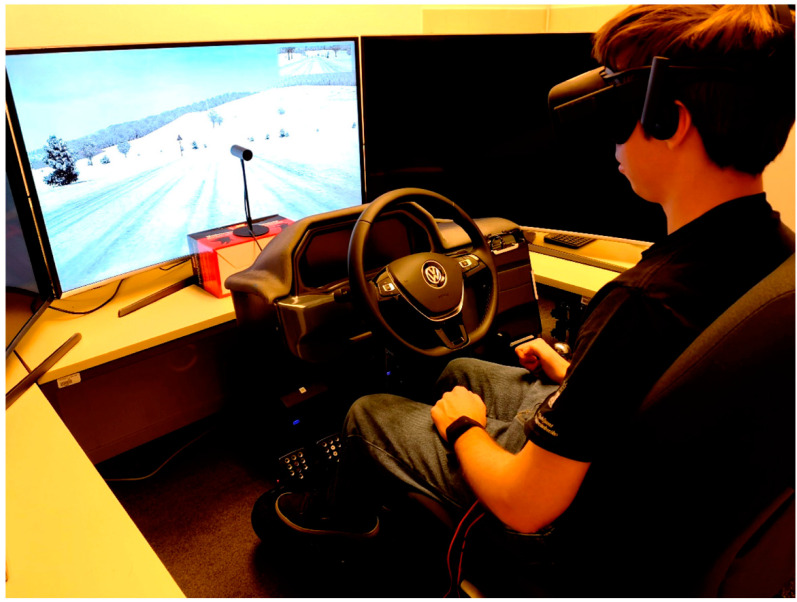
Illustration of the Nervtech driving simulator interior. Image is distributed under the Creative Commons Attribution License and taken from the study by Gruden et al. [4].

The study was performed in the Nervtech driving simulator (Nervtech d.o.o., Trzin, Slovenia) [34,35] with a motion platform with 4 degrees of freedom (yaw, pitch, roll, and heave) (Figure 1). The cockpit consisted of an adjustable car seat, a Fanatec ClubSport Wheel Base V2 steering wheel with dynamic feedback, and a Fanatec ClubSport Pedals V3 pedal set with three pedals (both from Fanatec, Endor AG, Landshut, Germany) [36,37]. The driving environment was simulated using the SCANeR simulation software (AV simulation, Boulogne, France) [38], a virtual reality headset (Oculus, Facebook Technologies LLC, Menlo Park, CA, USA) [39], and a stereo speaker set. Based on the definition of physical and functional fidelity as defined by Kinkade and Wheaton [40] and Hays [41], the simulator used in this study can be described as a high-fidelity driving simulator. After the participants were introduced to the experiment procedure, they signed informed consents in accordance with the Declaration of Helsinki and University Code of Ethics. The participants then completed a test trial to get acquainted with the experimental environment. They were instructed to raise their hand to stop the experiment at any time if sickness was too severe to be able to continue. The main part of the experiment was divided into three parts:

1. Baseline measurement before the driving simulation.
2. Driving simulation in autonomous vehicle.
3. EGG measurement while resting after driving simulation.

**Figure 2:**
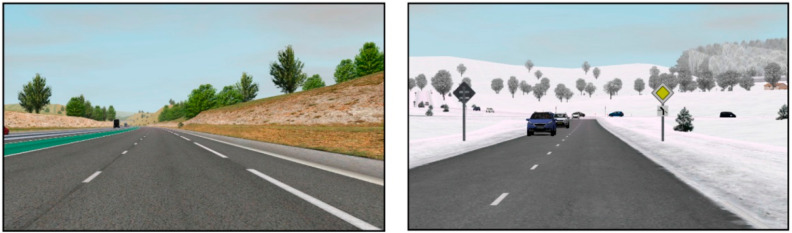
Screenshots of the driving scenarios employed in this study. (**Left**) highway with low traffic; (**Right**) country road with many vehicles and icy roads. Image is distributed under the Creative Commons Attribution License and taken from the study by Gruden et al. [4].

The driving simulation consisted of a less dynamic drive, which took part on a highway road and a more dynamic drive, which took part on a countryside road. The duration of each driving scene was approximately 7 min. The screenshots of the highway and countryside roads are given in Figure 2. The user study had a within-subject design as the participants’ state was measured before, during, and after AV driving simulation.

In order to induce higher levels of sickness and at the same time to reduce the potential and exaggerated EGG artifacts originating from motion or steering, fully autonomous driving was used (SAE level 5) [42]. The participants’ primary task was, therefore, only to observe the autonomous driving.

Five Ag/AgCl surface electrodes (H92SG, Kendall/Covidien, Dublin, Ireland) were placed on the participants’ stomach to measure three-channel EGG following the recommendations [33,43,44]. The EGG signal was recorded using an amplification and filtering device [10] and digitized using Biopac UIM100C MP150 Analog-to-Digital Converter (ADC) (Biopac Systems, Goleta, CA, USA) [45]. The sampling frequency was set to 2 Hz and the resolution was 16 bits.

### 2.2. EGG Preprocessing and Creation of Semi-Synthetic EGG Dataset

Preprocessing and feature extraction are performed offline in Matlab version R2019b (The Mathworks, Natick, MA, USA). For feature extraction, we used four segments corresponding to the baseline recording, countryside driving, highway driving, and EGG measurement after driving simulation while resting. Furthermore, participants were instructed to continuously report any experience of nausea or sickness by pressing a button, which was recorded alongside EGG data. As the frequency of EGG slow waves is approximately 3 cycles per minute, and therefore, a large segment of data is required to reliably detect the parameters, the nausea incidence variable was set for each segment to 1 for a segment if at least one button press was detected in that segment; otherwise, it was set to 0.

In the previous study examining noise effect on EGG analysis [46], SNRs from −50 dB to 15 dB with step 5 dB were used, but the majority of changes are seen from −20 dB to 15 dB. Hence, we created a semi-synthetic dataset by adding pseudo-random noise matching SNR values of −20 dB, −10 dB, 0 dB, 10 dB, and 20 dB. We report on exact mean SNRs as we are not able to keep SNRs at a constant level due to the application of a pseudo-random generator and the fact that noise power was set before the filtering which removed the majority of the wideband noise. The actual obtained SNRs in this study are −23 dB, −13 dB, −3 dB, 7 dB, and 17 dB.

Semi-synthetic EGG signals are digitally filtered with the 6th order zero-phase-distortion Butterworth bandpass filter with cutoff frequencies set at 1 cycle per minute (cpm) and 10 cpm (0.0167 Hz and 0.167 Hz). This process colored the noise by filtering it in a range from 1 cpm to 10 cpm. Due to the relatively low EGG amplitude (in the range of µV), the raw EGG signal is very sensitive to noises and motion artifacts. The recorded EGG signals are therefore inspected by an experienced researcher who removed parts with identified motion artifacts. Following an established procedure, these artifacts were identified as short, high-amplitude spikes in contrast to a slowly changing, low-amplitude signal representing gastric activity [4,10,22,47]. From the three measured EGG channels, the channel with the fewest artifacts is selected for further analysis. As 3 subjects are excluded as a consequence of low signal quality, the remaining analysis is performed in 17 subjects [4]. Succeeding feature extraction and nausea detection procedures are completely automated.

### 2.3. Automated Procedure for EGG-Based Features Extraction

All extracted EGG-based parameters are presented in Table 1. For the calculation of the level of randomness, we use Sample Entropy (SampEnt) of time series (SampEntT) and of Power Spectral Density (PSD) (SampEntP), as well as Spectral Entropy (SpectEnt). Additionally, we calculate the autocorrelation zero-crossing, as well as geometrical features corresponding to the Poincaré plots (SD1, SD2, and SDEGG in Table 1).

By visual inspection of SampEntT for embedding dimensions *m* = 2, 3, and 4, we conclude that there are two distinct groups with entropy higher and lower than 10. We use this information as empirical reasoning for splitting the data into two groups and for transforming SampEntT into categorical data for further analysis.

### 2.4. Statistical Analysis and Machine Learning Approach

For statistical analysis and machine learning, we use R programming language v4.1.2 [51] in R Studio environment (R Studio, Inc., Boston, MA, USA) with the following R packages from the Comprehensive R Archive Network (CRAN): dplyr [52], e1071 [53], caret [54], randomForest with randomForestExplainer [55], ggplot2 [56], effsize [57], and pROC [58]. For the sake of computational reproducibility, we shared R code and tables with EGG-based parameters on Zenodo repository [59].

To observe the impact of noise on the selected set of parameters, we statistically compare the values of calculated parameters in different SNR conditions. For normally distributed data, paired sample *t*-test is used to evaluate the existence of statistically significant differences between original and noisy EGG-based parameters for all SNRs, while Cohen’s *d* (*Cd*) is used to estimate the effect size. For non-normally distributed data, paired Wilcoxon’s Signed-Ranks test is used along with Cliff’s *delta* (*Cdelta*) as effect size measure. To test normality of the data, we use Shapiro–Wilk’s normality test with *p* set to 0.05.

Statistically significant differences among parameters corresponding to the nausea occurrence with those revealing the non-nausea occurrence are explored by *t*-test for normally distributed data, while non-parametric Wilcoxon–Mann–Whitney’s U test is used to compare dependent variables for two independent groups for non-normally distributed data. Here, we also calculated *Cd* and *Cdelta* for normally and non-normally distributed data, respectively.

Summary statistics for categorical entropy parameters (SampEntT) in relation to nausea occurrence is reported in conjunction with Pearson’s Chi square test with a simulated *p* value that is used to explore the significant correlation of nausea incidence within the categorical features. If not stated otherwise, the level for statistical significance is set at 0.05.

Binary Random Forest (RF) algorithm is constructed with the aim of classifying EGG-based parameters obtained from EGG data recorded with and without nausea occurrence. The choice of hyperparameters is based on the previous publication on a similar dataset classifying the physiological data [50] and on the Breiman and Cutler’s Random Forests for Classification and Regression [60,61]. The seed is set to 100 for the sake of reproducibility. The rationale for RF selection is that previous studies focused on the exploration of noise effect on classification performance, which showed that RF is relatively resistant to noise probably due to the bagging ensemble procedure [62,63]. All parameters from Table 1 are used as input to the RF classifier. We perform leave-one-out cross-validation on the training data as this type of validation is more suitable for datasets with a smaller number of instances [64]. The data are split into training (75%) and test sets (25%) taking into account the distribution balance within the splits by createDataPartition Caret procedure [54] to ensure that both training and test sets are representative of the dataset. Furthermore, we use the automated Caret procedure for tuning the classifier parameters.

The Caret procedure for RF resampling across tuning parameters resulted in the determination of optimal parameters termed mtry that corresponds to the number of variables randomly sampled at each data split. Accuracy is used by the automatic procedure to determine the optimal mtry by using the largest value of accuracy. For parameters obtained from noisy data, we apply two types of RF classifiers: (1) RF trained and tested on noisy data and (2) RF trained on original and tested on noisy data. Together with reported parameters for machine learning (ML) evaluation (Kappa, confidence interval for 95%, accuracy, sensitivity, specificity, precision, AUC (Area Under the Receiver Operating Curve) for both training and tests sets, and recall), we present feature importance plots. Features are ranked according to the score obtained by the sum of the number of times the feature is selected by all trees in created binary RF.

## 3. Results

Table 2 presents the results of the statistical analysis for the EGG-based parameters obtained on noisy data with different SNR levels in comparison to the parameters without synthetically added colored noise.

Comparative results of binary RF classification for original and noisy data are shown in Table 3. Evaluation ML parameters for classifiers trained on the original dataset and tested in noisy data are presented in Table 4. Results for RF classifiers with categorical SampEntT parameters for *m* = 2, 3, and 4 did not influence the results, so we decided not to present them.

For the original dataset, the mtry = 17 is selected by automatic tuning procedure with an accuracy of 0.790. For noisy datasets, mtry is set to 2 with an accuracy of 0.782, 17 with an accuracy of 0.827, 2 with an accuracy of 0.827, 17 with an accuracy of 0.827, and 2 with an accuracy of 0.810 for SNRs of −23 dB, −13 dB, −3 dB, 7 dB, and 17 dB, respectively.

**Figure 3:**
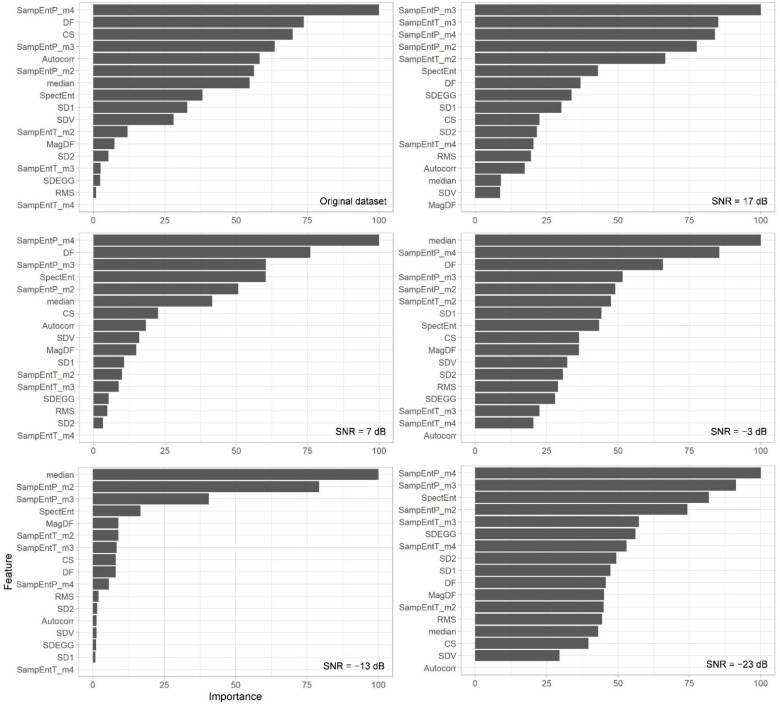
Importance plots for original and noisy datasets with different SNRs as a result of RF binary classifier for nausea detection. SNR stands for Signal-to-Noise Ratio.

Importance plots for RF applied on parameters obtained from noisy and original EGG data are presented in Figure 3.

The results of the *t*-tests for normally distributed data and of the Wilcoxon–Mann–Whitney’s U tests for non-normally distributed data are given in Table 5. Table 6 presents summary statistics for categorical SampEntT EGG-based parameters in relation to nausea occurrence.

Pearson’s Chi square test is used to explore the correlation of nausea occurrence with categorical SampEntT parameters for *m* = 2, 3, and 4, and for SNR = 17 dB, 7 dB, −3 dB, −13 dB, and −23 dB. However, no statistically significant relationships are found.

## 4. Discussion

As expected, noise influences EGG signals and consequently EGG-based parameters [66]. Presented results suggest that EGG-based parameters have divergent robustness to the additive colored noise. Here, we provide a quantitative approach to evaluate the noise effect on the results of statistical analysis and RF performance for sickness-related nausea detection during automated driving simulation. The discussion on different levels of SNR should be taken with precaution as the original dataset already contains a certain level of noise in the studied frequency band (from 1 cpm to 10 cpm). Hence, we stress that reported SNRs of semi-synthetic EGG data are lower than the actual SNRs. Taking into account that we applied channel selection and manual deletion of segments with exaggerated noise following the procedure applied in [4], we may assume that the true SNRs are non-significant in comparison to the reported SNRs.

Our results provide some important and intriguing insights into the behavior of selected EGG parameters in noisy conditions demonstrating their usability to detect EGG signals affected by nausea and simulator sickness. At the same time, we are aware that a larger and more diverse sample should be used to confirm or contradict the following insights obtained from the presented results.

### 4.1. Effect of Noise on EGG-Based Parameters

Expectedly, changing SNRs have different impacts on EGG-based parameters as their robustness to noise often degraded. Luckily, some features showed independent relationships with different SNRs. We therefore discuss each observed feature independently and indicate its potential and limitations in different noisy conditions.

Table 2 reveals that SampEntP is the least affected and does not change significantly across SNRs ranging from 17 dB to −23 dB, whereas SampEntT shows sensitivity to noise at higher SNRs (−13 dB, −23 dB). This may be due to the fact that the PSD of colored noise should be relatively flat on a studied segment of EGG spectrum (and thus relatively deterministic), whereas the noise signal in time domain is random. The sensitivity of SampEntT to noise at higher SNR values is to be expected as the substantial noise compromises the calculations of sample entropy [24].

Both SampEntT and SampEntP remain unchanged for all three embedding dimensions *m* indicating that the choice of *m* (if kept small) has no effect on SampEntP sensitivity to the noise in EGG signal. Moreover, SampEntP is the only parameter that had effect size *Cdelta* under 0.2 indicating negligible changes for all noise levels (Table 2). In all parameters, *Cdelta* and *Cd* where applicable degraded, i.e., revealed increased difference for increased noise levels.

Autocorrelation zero-crossing and median frequency did not change significantly for positive SNRs. This is expected as the calculation of autocorrelation and median frequency depends on the signal power which is in cases of SNR > 0 dB larger than the power of noise. On the other hand, DF remained stable in noisy conditions down to SNR = −3 dB, and unlike autocorrelation zero-crossing and median frequency, DF depends on just one peak location which could remain the same even in cases when noise power is larger than the signal power as long as it is not larger than the DF peak. SDV is not affected only at SNR of 17 dB, and this is expected as the variability of the signal is not the same as the variability of colored noise. Hence, SDV is easily affected, especially for lower SNRs.

Other parameters (RMS, MagDF, CS, SD1, SD2, and SDEGG) are influenced significantly for all SNR levels. Partly, this is expected as RMS and MagDF depend on the amplitude of the EGG signal which changes with added noise. SD1, SD2, and SDEGG depend on the relation between consecutive EGG samples, which is obviously highly sensitive to the colored pseudo-random noise. What is somewhat surprising is the fact that even relatively low noise levels (SNRs of 17 dB and 7 dB) had such a significant influence on these parameters.

### 4.2. Effect of Noise on Random Forest Classifier for Nausea Detection

Accuracy for the binary RF classifier is satisfactory (>88%). This is almost twice as better than a result presented by Dennison et al. [67] where the unimodal classifier trained only on two EGG features (percentages of band power of slow and fast stomach activity) reached an accuracy of 48.52% for four-scale sickness severity assessment. On the other hand, Dennison et al. [67] reported an accuracy higher than 95% when heterogeneous sensor data or solely EEG features are fed to the classifier for nausea severity classification. Future work should definitely be focused on multi-modal data fusion and properly selected EGG-based features for even better classification accuracies. AUC showed a poor classification result (0.616 for training with the best result of 0.667 for test set) which is expected as classifiers have poor specificity of ≤0.333 (Table 3 and Table 4). AUC remained constant for the training set throughout all results, probably indicating less training confidence. Although the AUC for the test set reached a maximum of 0.667 and slightly outperformed on unseen instances in comparison to AUC on the training set (0.616), it still performed poorly. It could be argued that without higher specificity, the data cannot reveal a strong machine learning pattern. The classifier performance remained stable even for noisy datasets (Table 3). The highest degradation happened at the lowest SNRs of −17 dB and −7 dB as expected. This is in line with previously reported results indicating that with higher noise levels, RF performance degrades while it is reasonably resistant to the noise procedure [62,63]. The reason for this may be in the fact that the majority of EGG-based features changed statistically significantly for these noise levels (Table 2) and that non-linear relations among parameters probably changed. A similar result is seen when a classifier previously trained on original data is tested with a noisy dataset (Table 4), indicating that RF may be considered a good candidate for nausea detection in simulated automated vehicles by EGG-based parameters.

Class imbalance may be the problem with the available dataset. Although all applied methods should compensate for imbalance, they cannot eliminate it. For nausea occurrence, overall, 12 out of 68 (~17.6%) EGG segments had positive nausea incidence. We hypothesize that RF deals well with imbalanced data in comparison with other classifiers as it uses data bootstrapping by random sampling with replacement [60]. The problem with nausea occurrence is that although sensitivity/recall is high (100%)—meaning that all subjects with nausea are correctly classified—the specificity is low (33.3%) so the classifier is not good at discerning those without reported nausea. This may not be caused solely by the RF, as it may also be the consequence of subjective reporting of nausea occurrence. We do not exclude the case that some subjects probably failed to report sickness when it actually happened.

Importance plots should be taken with precaution due to the existence of cross-correlations among introduced parameters that can influence importance. Feature cross-correlations cannot influence the RF accuracy [68,69], indicating that differences in the importance plot originate from the SNR influence. Importance plots reveal that SampEntP rose to the top five with the highest importance, indicating that it may be one of the most relevant features (Figure 3). This is rather important as SampEntP could not differentiate between nausea and non-nausea data with a classical statistical approach (Table 5). The reason for this may be in the fact that the statistical test failed to detect non-linear relations in comparison to the ML algorithm.

Autocorrelation zero-crossing and CS appear only in the first five features in the importance plot for the original dataset, while SampEntT, DF, MagDF, and median also appear within the five most relevant features. Interestingly, RMS, SDV, and features derived from the Poincaré plot (SD1, SD2, and SDEGG) do not appear within the top five features in all importance plots incorporating original and semi-synthetic noisy EGG-based parameters (Figure 3). This may be the consequence of RF which fails to detect their influence or of the fact that the influence of parameters derived from the Poincaré plot is minor in comparison to other EGG-based parameters.

### 4.3. Effect of Noise on Detection of Nausea through Statistical Tests

SpectEnt shows the statistically significant difference between those with and without reported nausea (Table 5) for all positive SNR values. This is not in line with the results reported in Table 2 where this feature remained statistically changed for all SNR values. We can argue that the changes in the signals introduced by nausea occurrence are more dominant in comparison to changes introduced by noise, which makes these two parameters mildly robust to the added colored noise. Similarly, CS does not show any tolerance to noise, but its ability to discern among EGG with and without nausea occurrence is stable for a relatively low noise level (SNR = 17 dB). However, this is only true if *p* is set to 0.001 for testing normal distribution, but not if *p* is set to 0.05. Median frequency, DF, autocorrelation zero-crossing, as well as SampEntT and SampEntP for all embedding dimensions *m* show no statistically significant difference between those features with and without nausea occurrence for the original dataset. The difference that arose with added noise is probably merely coincidental or falsely produced as a result of additive synthetic noise.

DF is not affected by the nausea occurrence and *p* value is much lower than in the previous study that reported results on the same dataset [4]. However, these results cannot be directly compared as different independent variables and different statistical tests were used in the current study.

Transformation of SampEntT for three embedding dimensions *m* into categorical variables for original data and all noise levels did not produce any significant result yielding to a conclusion that SampEntT should be treated as a numerical variable. Table 6 speaks in favor of such a finding as reported proportions are indecisive for the original data, and what is more convincing is that categorical SampEntT appear very sensitive to SNR levels, which is contrasting to the SampEntT robustness for all embedding dimensions *m* from Table 2.

Effect size reported by *Cd* and *Cdelta* tended to decrease with higher SNRs (Table 5) meaning that the higher noise contamination influences parameter sensitivity to nausea occurrence. In all cases where statistically significant differences are reported in Table 5, absolute effect size parameters ranged from small (>0.2) to large differences (>0.8). In all other cases, differences were rather small (<0.4) or negligible (<0.2), except for the SpectEnt which, despite the large effect size (>0.6), did not reveal statistical significance for higher noise levels (−13 dB and −23 dB).

### 4.4. Limitations of the Study

Although we present a detailed analysis of EGG-based parameters for simulator nausea evaluation, we recognize the following limitations:

1. We use a discrete set of predefined SNRs, and one should note that the actual SNRs were much higher, as our data were already contaminated with noises and artifacts. Despite the linear Butterworth filtering applied in the preprocessing stage, the noise with overlapping frequency content probably remains present in the semi-synthetic EGG dataset. Future efforts towards the generation of synthetic noises would provide a firm basis for exact SNR contamination and more reliable analysis.
2. It should also be noted that sample entropy scaling parameter *r* is kept constant at the 0.15 of the noiseless data standard deviation. This value was determined empirically based on the recommendations [24]. Adjusting this value for different SNRs may have a further effect on the results and should be investigated in the future.
3. We apply procedures for automatic feature calculation. However, a guided visual observation and manual corrections are still considered a gold standard for the evaluation of EGG-based parameters especially in cases of excessive noises [10,70,71]. We use visual inspection only for channel selection. Despite this drawback, we obtained promising results in nausea assessment by both statistical and ML approaches.
4. We select the embedded dimension *m* for sample entropy calculation empirically. For future selection and discussion on embedding dimension selection, one may look at outstanding reasoning by Matilla-García et al. [72].
5. We did not apply unimodal or multi-modal machine learning algorithms, and we do not provide comparison of existing machine learning techniques as in [67].
6. Our method is applied only for nausea occurrence. Further customization of presented EGG-based parameters and complementary approach by RF and statistical analysis should yield at assessment of sickness levels similarly as in [67].
7. The dataset used for the analysis contains more male than female participants. However, we do not consider this to be a major drawback of our study, as we were not interested in the differences between the genders but focused on the relationships between the occurrence of nausea, the EGG parameters, and noise. Moreover, a systematic review performed by Grassini and Laumann [73] showed conflicting results in published studies focused on determining sex differences in experiencing simulator sickness.
8. We did not use multi biomarkers for the assessment of sickness occurrence as our focus was solely on the direct assessment of gastric activity. However, future studies should be focused on a promising heterogeneous approach as, for example, suggested by Dennison et al. [67].

## 5. Conclusions

The presented results highlighted the importance of appropriate EGG parameters selection when the higher levels of noise are anticipated during driving simulation for nausea detection. Although some EGG-based features are sensitive to the nausea occurrence, they may at the same time be sensitive to the higher noise levels. This is important for the study design of EGG-based nausea detection within driving simulators encompassing haptic frameworks.

Feature engineering and decision making by both machine learning and statistical tests may be fully automated for the future adoption of EGG-based nausea detection. These two approaches are complementary, as ML algorithms benefit from non-linear relations that cannot be revealed by statistical tests such as in the case of sample entropy parameters.

Sample entropy of EGG signals stands out among all other parameters due to its exceptional robustness to the colored noise and due to its ability to differentiate between EGG segments with and without nausea occurrence for signals recorded in the driving simulator. The potential of sample entropy to detect nausea in noisy EGG signals should be further explored in other EGG-related dynamic studies. The assessment of EGG signals with the sample entropy feature may open a door to the scientific experiments that were never conducted before as exaggerated EGG sensitivity to noises and artifacts may not present an obstacle anymore.

Sickness-related nausea detection in driving simulators by EGG-based parameters is an important aspect that could assist in the overall comfort improvement in both simulators and automated vehicles. This study emphasized the importance of proper EGG-based feature selection when dynamic and noisy EGG recording is anticipated (e.g., different levels of driving automation resulting in subject’s maneuvers interfacing vehicle commands or in-vehicle infotainment). Additionally, our results revealed the superiority of sample entropy in relation to other parameters and in combination with the RF ML algorithm.

## Figures and Tables

**Table 1:** 17 EGG-based features calculated from EGG segments recorded during a simulated drive in 17 subjects (SampEntT and SampEntP are separately evaluated for embedding dimensions *m* = 2, 3, and 4). The number of observations for all four segments is 17 × 4 = 68 and the total number of features for 17 subjects is 17 × 68 = 1156. PSD stands for Power Spectral Density.

Feature	Explanation	Unit	References
RMS (Root Mean Square)	RMS of the amplitude of EGG selected segment	μV	[4,10]
median	Median frequency of PSD of selected EGG segment	cpm (cycles per minute)	
DF (Dominant Frequency)	Dominant frequency of PSD of selected EGG segment	cpm	[4,10,48]
MagDF	Magnitude of DF in PSD of selected EGG segment	mV^2^/Hz	[4,10,48]
CS (Crest Factor)	CS of PSD of selected EGG segments	/	[4,10]
SDV (Spectral Variation Distribution)	PSD with magnitude higher than 25% of DF	%	[4]
SampEntT (Sample Entropy of Time Series)	Embedding dimensions *m* = 2, 3, and 4	/	Introduced here and inspired by [49]
SampEntP (Sample Entropy of PSD)	Embedding dimensions *m* = 2, 3, and 4	/	
SpectEnt (Spectral Entropy)	/	/	
Autocorr (Autocorrelation zero-crossing)	The first lag of autocorrelation function of EGG at which autocorrelation equals 0	S	Introduced here and inspired by [46]
SD1	Transverse line of the Poincaré plot in the perpendicular direction. A Poincaré plot presents a scatter plot of the current EGG sample in relation to the prior EGG sample.	μV	Introduced here and inspired by [31,50]
SD2	Longitudinal line of the Poincaré plot in the perpendicular direction.	μV	
SDEGG	Standard deviation of EGG samples obtained from the SD1 and SD2.	μV	

**Table 2:** Results of paired *t*-tests and Cohen’s *d* (for normally distributed data), as well as paired Wilcoxon’s Signed-Ranks tests and Cliff’s *delta* (for not normally distributed data) for comparison of non-noisy and noisy EGG-based parameters are presented. For testing normality, *p* was set at 0.05. Values with *p* > 0.05 (no significant difference due to the noise is observed, i.e., EGG-based features are robust to the noise levels) are highlighted in bold. SNR stands for Signal-to-Noise Ratio, *V* stands for V-statistics, *p* for probability, *Cdelta* for Cliff’s *delta*, *Cd* for Cohen’s *d*, *t* for t-statistics, and *df* for degrees of freedom.

Features	SNR = 17 dB	SNR = 7 dB	SNR = −3 dB	SNR = −13 dB	SNR = −23 dB
RMS	*V* = 321, *p* < 0.001, *Cdelta* = 0.021	*V* = 77, *p* < 0.001, *Cdelta* = 0.092	*V* = 0, *p* < 0.001, *Cdelta* = 0.344	*V* = 0, *p* < 0.001, *Cdelta* = 0.733	*V* = 0, *p* < 0.001, *Cdelta* = 0.951
median	***V* = 188.5,** ***p* = 0.750,** ***Cdelta* = 0.002**	***t* = −1.761,** ***df* = 67,** ***p* = 0.083,** ***Cd* = −0.060**	*t* = −2.852, *df* = 67, *p* = 0.006, *Cd* = −0.265	*t* = −4.5567, *df* = 67, *p* < 0.001, *Cd* = −0.652	*V* = 460, *p* < 0.001, *Cdelta* = −0.457
MagDF	*V* = 739, *p* = 0.008, *Cdelta* = −0.007	*V* = 812, *p* = 0.028, *Cdelta* = −0.031	*V* = 206, *p* < 0.001, *Cdelta* = −0.254	*V* = 0, *p* < 0.001, *Cdelta* = −0.653	*V* = 0, *p* < 0.001, *Cdelta* = −0.919
DF	***V* = 64,** ***p* = 0.476,** ***Cdelta* = 0.007**	***V* = 240,** ***p* = 0.461,** ***Cdelta* = −0.061**	***V* = 508.5,** ***p* = 0.730,** ***Cdelta* = −0.034**	*V* = 647, *p* = 0.009, *Cdelta* = −0.253	*V* = 845, *p* = 0.045, *Cdelta* = −0.176
CS	*V* = 1591, *p* = 0.011, *Cdelta* = 0.031	*V* = 1688, *p* = 0.002, *Cdelta* = 0.098	*V* = 1872, *p* < 0.001, *Cdelta* = 0.246	*V* = 2108, *p* < 0.001, *Cdelta* = 0.533	*V* = 2136, *p* < 0.001, *Cdelta* = 0.546
SDV	***V* = 495,** ***p* = 0.342,** ***Cdelta* = −0.012**	*V* = 335, *p* < 0.001, *Cdelta* = −0.135	*V* = 344.5, *p* < 0.001, *Cdelta* = −0.344	*V* = 173.5, *p* < 0.001, *Cdelta* = −0.674	*V* = 97.5, *p* < 0.001, *Cdelta* = −0.764
SampEntT_m2	***V* = 920,** ***p* = 0.46,** ***Cdelta* = 0.031**	***V* = 610,** ***p* = 0.256,** ***Cdelta* = −0.052**	***V* = 1213,** ***p* = 0.249,** ***Cdelta* = 0.085**	*V* = 1918, *p* < 0.001, *Cdelta* = 0.413	*V* = 2330, *p* < 0.001, *Cdelta* = 0.754
SampEntT_m3	***V* = 626.5,** ***p* = 0.350,** ***Cdelta* = 0.067**	***V* = 444,** ***p* = 0.556,** ***Cdelta* = −0.018**	***V* = 787,** ***p* = 0.083,** ***Cdelta* = 0.136**	*V* = 1666, *p* < 0.001, *Cdelta* = 0.410	*V* = 2285, *p* < 0.001, *Cdelta* = 0.682
SampEntT_m4	***V* = 254,** ***p* = 0.914,** ***Cdelta* = 0.039**	***V* = 221,** ***p* = 0.948,** ***Cdelta* = 0.018**	***V* = 437,** ***p* = 0.338,** ***Cdelta* = 0.101**	*V* = 1598, *p* < 0.001, *Cdelta* = 0.419	*V* = 2209, *p* < 0.001, *Cdelta* = 0.671
SampEntP_m2	***V* = 1289,** ***p* = 0.480,** ***Cdelta* = 0.030**	***V* = 1349,** ***p* = 0.284,** ***Cdelta* = 0.040**	***V* = 1278,** ***p* = 0.523,** ***Cdelta* = −0.006**	***V* = 1373,** ***p* = 0.223,** ***Cdelta* = 0.030**	***V* = 1221,** ***p* = 0.772,** ***Cdelta* = 0.008**
SampEntP_m3	***V* = 1306,** ***p* = 0.418,** ***Cdelta* = 0.015**	***V* = 1252,** ***p* = 0.631,** ***Cdelta* = 0.006**	***V* = 1275,** ***p* = 0.535,** ***Cdelta* = −0.013**	***V* = 1369,** ***p* = 0.232,** ***Cdelta* = 0.083**	***V* = 1086,** ***p* = 0.597,** ***Cdelta* = −0.042**
SampEntP_m4	***V* = 1293,** ***p* = 0.465,** ***Cdelta* = 0.008**	***V* = 1344,** ***p* = 0.297,** ***Cdelta* = −0.009**	***V* = 1417,** ***p* = 0.137,** ***Cdelta* = 0.020**	***V* = 1385,** ***p* = 0.196,** ***Cdelta* = 0.047**	***V* = 955,** ***p* = 0.184,** ***Cdelta* = −0.154**
SpectEnt	*V* = 727, *p* = 0.006, *Cdelta* = −0.019	*V* = 340, *p* < 0.001, *Cdelta* = −0.154	*V* = 180, *p* < 0.001, *Cdelta* = −0.416	*V* = 156, *p* < 0.001, *Cdelta* = −0.695	*V* = 161, *p* < 0.001, *Cdelta* = −0.724
Autocorr	***V* = 30,** ***p* = 0.351,** ***Cdelta* = 0.024**	***V* = 27,** ***p* = 0.608,** ***Cdelta* = 0.027**	*V* = 540, *p* < 0.001, *Cdelta* = 0.245	*V* = 1066, *p* < 0.001, *Cdelta* = 0.492	*V* = 1215, *p* < 0.001, *Cdelta* = 0.557
SD1	*V* = 205, *p* < 0.001, *Cdelta* = −0.021	*V* = 19, *p* < 0.001, *Cdelta* = −0.096	*V* = 0, *p* < 0.001, *Cdelta* = −0.384	*V* = 0, *p* < 0.001, *Cdelta* = −0.766	*V* = 0, *p* < 0.001, *Cdelta* = −0.958
SD2	*V* = 327, *p* < 0.001, *Cdelta* = −0.022	*V* = 80, *p* < 0.001, *Cdelta* = −0.093	*V* = 0, *p* < 0.001, *Cdelta* = −0.345	*V* = 0, *p* < 0.001, *Cdelta* = −0.733	*V* = 0, *p* < 0.001, *Cdelta* = −0.951
SDEGG	*V* = 321, *p* < 0.001, *Cdelta* = −0.021	*V* = 76, *p* < 0.001, *Cdelta* = −0.093	*V* = 0, *p* < 0.001, *Cdelta* = −0.344	*V* = 0, *p* < 0.001, *Cdelta* = −0.733	*V* = 0, *p* < 0.001, *Cdelta* = −0.951

**Table 3:** ML evaluation parameters for binary RF classification of nausea for original and noisy data. The best results are presented in bold. SNR stands for Signal-to-Noise Ratio, CI stands for Confidence Interval, and AUC stands for Area Under the Curve. Two AUC values are presented for the validation set and for the test set ^1^.

**Evaluation** **Classifier** **Metrics**	**Original** **Dataset**	**Noisy Data**				
		**SNR = 17 dB**	**SNR = 7 dB**	**SNR = −3 dB**	**SNR = −13 dB**	**SNR = −23 dB**
Kappa	**0.452**	**0.452**	0.301	**0.452**	−0.214	−0.097
95% CI	**(0.636, 0.985)**	**(0.636, 0.985)**	(0.566, 0.962)	**(0.636, 0.985)**	(0.383, 0.858)	(0.501, 0.932)
Accuracy	**0.882**	**0.882**	0.823	**0.882**	0.647	0.765
Sensitivity	**1.000**	**1.000**	0.929	**1.000**	0.786	0.929
Specificity	**0.333**	**0.333**	**0.333**	**0.333**	0	0
Precision	**0.875**	**0.875**	0.867	**0.875**	0.786	0.812
Recall	**1.000**	**1.000**	0.929	**1.000**	0.786	0.929
AUC (training)	**0.616**	**0.616**	**0.616**	**0.616**	**0.616**	**0.616**
AUC (test)	**0.667**	**0.667**	0.631	**0.667**	0.393	0.464

^1^ As suggested by reviewer and literature [64], we applied leave-one-out cross validation. However, the leave-one-out validation performed only slightly better than 10-fold cross-validation (SNR = −13 dB in Table 3 and SNR = −23 dB in Table 4). This may be the result of similar instances in a dataset which did not have larger effect on separate models (in cases when similar instances are omitted). The discussion on selection of cross-validation procedures is out of scope of this paper and it has been discussed elsewhere [65].

**Table 4:** ML evaluation parameters for binary RF trained and validated on original dataset, and further tested on noisy data with different noise levels. The best results are presented in bold. SNR stands for Signal-to-Noise Ratio, CI stands for Confidence Interval, and AUC stands for Area Under the Curve. Two AUC values are presented for the validation set and for the test set.

Evaluation Classifier Metrics	Noisy Test Data				
	SNR = 17 dB	SNR = 7 dB	SNR = −3 dB	SNR = −13 dB	SNR = −23 dB
Kappa	**0.452**	**0.452**	**0.452**	0	0
95% CI	**(0.636, 0.985)**	**(0.636, 0.985)**	**(0.636, 0.985)**	(0.566, 0.962)	(0.566, 0.962)
Accuracy	**0.882**	**0.882**	**0.882**	0.823	0.823
Sensitivity	**1.000**	**1.000**	**1.000**	1.000	1.000
Specificity	**0.333**	**0.333**	**0.333**	0	0
Precision	**0.875**	**0.875**	**0.875**	0.823	0.823
Recall	**1.000**	**1.000**	**1.000**	1.000	1.000
AUC (training)	**0.616**	**0.616**	**0.616**	**0.616**	**0.616**
AUC (test)	**0.667**	**0.667**	**0.667**	0.500	0.500

**Table 5:** Results of statistical tests for the comparison of parameters with and without nausea for non-noisy and noisy data with different noise levels. For testing normality, *p* is set at 0.05. Statistically significant differences are presented in bold (*p* < 0.05). SNR stands for Signal-to-Noise Ratio, *W* stands for W-statistics, *p* for probability, *Cdelta* for Cliff’s *delta*, *Cd* for Cohen’s *d*, *t* for t-statistics, and *df* for degrees of freedom.

Features	Original	SNR = 17 dB	SNR = 7 dB	SNR = −3 dB	SNR = −13 dB	SNR = −23 dB
RMS	*W* = 245, *p* = 0.145, *Cdelta* = −0.270	*W* = 247, *p* = 0.154, *Cdelta* = −0.265	*W* = 243, *p* = 0.137, *Cdelta* = −0.277	*W* = 256, *p* = 0.201, *Cdelta* = −0.238	*W* = 297, *p* = 0.536, *Cdelta* = −0.116	*W* = 299, *p* = 0.557, *Cdelta* = −0.110
median	*t* = −0.8408, *df* = 19.075, *p* = 0.411, *Cd* = −0.232	*W* = 268, *p* = 0.277, *Cdelta* = −0.202	*t* = −1.7105, *df* = 18.665, *p* = 0.104, *Cd* = −0.480	*t* = −1.7556, *df* = 14.249, *p* = 0.101, *Cd* = −0.641	***t* = −2.97,** ***df* = 18.279,** ***p* = 0.008,** ***Cd* = −0.846**	*W* = 246, *p* = 0.150, *Cdelta* = −0.268
MagDF	*W* = 261, *p* = 0.231, *Cdelta* = −0.223	*W* = 263, *p* = 0.243, *Cdelta* = −0.217	*W* = 263, *p* = 0.243, *Cdelta* = −0.217	*W* = 263, *p* = 0.243, *Cdelta* = −0.217	*W* = 301, *p* = 0.579, *Cdelta* = −0.104	*W* = 317, *p* = 0.766, *Cdelta* = −0.056
DF	*W* = 319.5, *p* = 0.796, *Cdelta* = −0.049	*W* = 293.5, *p* = 0.498, *Cdelta* = −0.126	*W* = 245.5, *p* = 0.147, Cdelta = −0.269	***W* = 191,** ***p* = 0.020,** ***Cdelta* = −0.431**	*W* = 296.5, *p* = 0.530, *Cdelta* = −0.117	*W* = 315.5, *p* = 0.747, *Cdelta* = −0.061
CS	***W* = 469,** ***p* = 0.033,** ***Cdelta* = 0.396**	***W* = 468,** ***p* = 0.034,** ***Cdelta* = 0.393**	*W* = 408, *p* = 0.250, *Cdelta* = 0.214	*W* = 377, *p* = 0.515, *Cdelta* = 0.122	*W* = 343, *p* = 0.917, *Cdelta* = 0.021	*W* = 385, *p* = 0.435, *Cdelta* = 0.146
SDV	***t* = −2.7527,** ***df* = 36.441,** ***p* = 0.009,** ***Cd* = −0.559**	*W* = 252, *p* = 0.179, *Cdelta* = −0.250	*W* = 266, *p* = 0.263 ^2^, *Cdelta* = −0.208	*W* = 353, *p* = 0.791, *Cdelta* = 0.050	*W* = 287.5, *p* = 0.439, *Cdelta* = −0.144	*W* = 308.5, *p* = 0.663, *Cdelta* = −0.082
SampEntT_m2	*W* = 421, *p* = 0.174, *Cdelta* = 0.253	***W* = 486.5,** ***p* = 0.016,** ***Cdelta* = 0.448**	*W* = 443.5, *p* = 0.085, *Cdelta* = 0.320	*W* = 411.5, *p* = 0.228, *Cdelta* = 0.225	*W* = 393.5, *p* = 0.359, *Cdelta* = 0.171	*W* = 372, *p* = 0.568, *Cdelta* = 0.107
SampEntT_m3	*W* = 415.5, *p* = 0.198, *Cdelta* = 0.237	***W* = 475,** ***p* = 0.025,** ***Cdelta* = 0.414**	*W* = 440, *p* = 0.091, *Cdelta* = 0.309	*W* = 406.5, *p* = 0.259, *Cdelta* = 0.210	*W* = 421, *p* = 0.174, *Cdelta* = 0.253	*W* = 388, *p* = 0.407, *Cdelta* = 0.155
SampEntT_m4	*W* = 420, *p* = 0.136, *Cdelta* = 0.250	***W* = 459.5,** ***p* = 0.034,** ***Cdelta* = 0.367**	*W* = 446, *p* = 0.054, *Cdelta* = 0.327	*W* = 399.5, *p* = 0.293, *Cdelta* = 0.189	*W* = 382, *p* = 0.464, *Cdelta* = 0.137	*W* = 370, *p* = 0.590, *Cdelta* = 0.101
SampEntP_m2	*W* = 413, *p* = 0.218, *Cdelta* = 0.229	*W* = 438, *p* = 0.102, *Cdelta* = 0.303	*W* = 406, *p* = 0.263, *Cdelta* = 0.208	*W* = 420, *p* = 0.179, *Cdelta* = 0.250	***W* = 504,** ***p* = 0.007,** ***Cdelta* = 0.500**	*W* = 256, *p* = 0.201, *Cdelta* = −0.238
SampEntP_m3	*W* = 408, *p* = 0.250, *Cdelta* = 0.214	*W* = 404, *p* = 0.277, *Cdelta* = 0.202	*W* = 401, *p* = 0.299, *Cdelta* = 0.193	*W* = 404, *p* = 0.277, *Cdelta* = 0.202	***W* = 525,** ***p* = 0.002,** ***Cdelta* = 0.562**	*W* = 273, *p* = 0.315, *Cdelta* = −0.187
SampEntP_m4	*W* = 410, *p* = 0.237, *Cdelta* = 0.220	*W* = 416, *p* = 0.201, *Cdelta* = 0.238	*W* = 442, *p* = 0.090, *Cdelta* = 0.315	***W* = 466,** ***p* = 0.037,** ***Cdelta* = 0.387**	*W* = 427, *p* = 0.145, *Cdelta* = 0.271	*W* = 339, *p* = 0.968, *Cdelta* = 0.009
SpectEnt	***W* = 172,** ***p* = 0.008,** ***Cdelta* = −0.488**	***W* = 175,** ***p* = 0.010,** ***Cdelta* = −0.479**	***W* = 160,** ***p* = 0.005,** ***Cdelta* = −0.524**	*W* = 226, *p* = 0.078, *Cdelta* = −0.327	*t* = −2.032, *df* = 17.237, *p* = 0.058, *Cd* = −0.606	*t* = −2.055, *df* = 17.409, *p* = 0.055, *Cd* = −0.608
Autocorr	*W* = 439, *p* = 0.084, *Cdelta* = 0.306	*W* = 438.5, *p* = 0.082, *Cdelta* = 0.305	*W* = 447.5, *p* = 0.060, *Cdelta* = 0.332	***W* = 446.5,** ***p* = 0.047,** ***Cdelta* = 0.329**	*W* = 399, *p* = 0.261, *Cdelta* = 0.187	*W* = 369, *p* = 0.548, *Cdelta* = 0.098
SD1	*W* = 232, *p* = 0.096, *Cdelta* = −0.309	*W* = 232, *p* = 0.096, *Cdelta* = −0.309	*W* = 231, *p* = 0.093, *Cdelta* = −0.312	*W* = 249, *p* = 0.164, *Cdelta* = −0.259	*W* = 285, *p* = 0.417, *Cdelta* = −0.152	*W* = 294, *p* = 0.504, *Cdelta* = −0.125
SD2	*W* = 245, *p* = 0.145, *Cdelta* = −0.271	*W* = 246, *p* = 0.150, *Cdelta* = −0.268	*W* = 243, *p* = 0.137, *Cdelta* = −0.277	*W* = 257, *p* = 0.207, *Cdelta* = −0.235	*W* = 298, *p* = 0.546, *Cdelta* = −0.113	*W* = 299, *p* = 0.557, *Cdelta* = −0.110
SDEGG	*W* = 245, *p* = 0.145, *Cdelta* = −0.271	*W* = 247, *p* = 0.154, *Cdelta* = −0.265	*W* = 243, *p* = 0.137, *Cdelta* = −0.277	*W* = 256, *p* = 0.201, *Cdelta* = −0.238	*W* = 297, *p* = 0.536, *Cdelta* = −0.116	*W* = 299, *p* = 0.557, *Cdelta* = −0.110

^2^ SDV for SNR = 7 dB and 17 dB appears statistically significant if *p* is set to 0.05 for testing normal distribution. All other parameters throughout the manuscript remained the same for *p* set to 0.001. We decided to report more rigor results.

**Table 6:** Summary statistics for categorical data. For more information, please see Materials and Methods section. SNR stands for Signal-to-Noise Ratio.

Feature	Proportions of Reported Nausea Corresponding to the Selected Feature (Low/High)						Proportions of Regular EGG Corresponding to the Selected Features (Low/High)					
	Original	SNR = 17 dB	SNR = 7 dB	SNR = −3 dB	SNR = −13 dB	SNR = −23 dB	Original	SNR = 17 dB	SNR = 7 dB	SNR = −3 dB	SNR = −13 dB	SNR = −23 dB
SampEntT_m2	0.58/0.25	0.58/0.17	0.50/0.25	0.42/0.25	0.67/0.08	0.92/0.00	0.27/0.30	0.21/0.25	0.23/0.32	0.25/0.12	0.70/0.07	0.93/0.00
SampEntT_m3	0.50/0.42	0.75/0.25	0.67/0.33	0.67/0.33	0.75/0.17	0.92/0.00	0.37/0.61	0.39/0.55	0.41/0.59	0.48/0.43	0.84/0.09	0.98/0.02
SampEntT_m4	0.33/0.58	0.50/0.50	0.42/0.58	0.50/0.50	0.67/0.33	0.92/0.08	0.30/0.70	0.25/0.71	0.29/0.70	0.36/0.62	0.70/0.25	0.95/0.05

## Data Availability

The raw data presented in this study and Matlab code for feature extraction are available on request from the corresponding author. R code and CSV tables with relevant EGG-based parameters are available on Zenodo repository and shared under GNU General Public License [59].
